# Protocol for the development of a core outcome set for lateral elbow tendinopathy (COS-LET)

**DOI:** 10.1186/s13063-021-05291-9

**Published:** 2021-05-10

**Authors:** Marcus Bateman, Jonathan P. Evans, Viana Vuvan, Val Jones, Adam C. Watts, Joideep Phadnis, Leanne Bisset, Bill Vicenzino

**Affiliations:** 1 Derby Shoulder Unit, Royal Derby Hospital, University Hospitals of Derby & Burton NHS Foundation Trust, Uttoxeter Road, Derby, UK; 2 Health Services and Policy Research, Smeall Building, JS03, St Lukes Campus, Exeter, UK; 3 School of Health and Rehabilitation Sciences: Physiotherapy, The University of Queensland, St Lucia, Brisbane, Australia; 4 Sheffield Shoulder and Elbow Unit, Sheffield Teaching Hospitals NHS Foundation Trust, Sheffield, UK; 5 Wrightington, Wigan and Leigh Teaching Hospitals NHS Foundation Trust, Wrightington Hospital, Hill Lane, Wigan, UK; 6 University Hospitals Sussex NHS Foundation Trust, Royal Sussex County Hospital, Eastern Road, Brighton, UK; 7 Brighton & Sussex Medical School, University of Sussex, 94 N - S Rd, Falmer, Brighton, UK; 8 Menzies Health Institute Queensland, Griffith University, Gold Coast, Australia

**Keywords:** Lateral elbow tendinopathy, Tennis elbow, Core outcome set

## Abstract

**Background:**

Lateral elbow tendinopathy (LET) is a common condition that can cause significant disability and associated socioeconomic cost. Although it has been widely researched, outcome measures are highly variable which restricts evidence synthesis across studies. In 2019, a working group of international experts, health care professionals and patients, in the field of tendinopathy (International Scientific Tendinopathy Symposium Consensus (ICON) Group), published the results of a consensus exercise defining the nine core domains that should be measured in tendinopathy research. The aim of this study is to develop a core outcome set (COS) for LET mapping to these core domains. The primary output will provide a template for future outcome evaluation of LET. In this protocol, we detail the methodological approach to the COS-LET development.

**Methods:**

This study will employ a three-phase approach. (1) A systematic review of studies investigating LET will produce a comprehensive list of all instruments currently employed to quantify the treatment effect or outcome. (2) Instruments will be matched to the list of nine core tendinopathy outcome domains by a Steering Committee of clinicians and researchers with a specialist interest in LET resulting in a set of candidate instruments. (3) An international three-stage Delphi study will be conducted involving experienced clinicians, researchers and patients. Within this Delphi study, candidate instruments will be selected based upon screening using the Outcome Measures in Rheumatology (OMERACT) truth, feasibility and discrimination filters with a threshold of 70% agreement set for consensus.

**Conclusions:**

There is currently no COS for the measurement or monitoring of LET in trials or clinical practice. The output from this project will be a minimum COS recommended for use in all future English language studies related to LET. The findings will be published in a high-quality journal and disseminated widely using professional networks, social media and via presentation at international conferences.

**Trial registration:**

Registered with the Core Outcome Measures in Effectiveness Trials (COMET) database, November 2019. https://www.comet-initiative.org/Studies/Details/1497.

## Key points

- There is a wide variability in the outcome measures used in lateral elbow tendinopathy research.
- This protocol outlines the methodology used to derive a standardised set of validated outcome measures.
- The core outcome set for lateral elbow tendinopathy (COS-LET) will provide guidance on the minimum recommended outcomes to be used in future research, which, if implemented broadly, will better represent the core domains of tendinopathy and assist in the future systematic review and meta-analysis.

## Background and objectives

Pain arising from the tendons on the lateral side of the elbow is common in adults, particularly in middle age [1]. Historically, it has been known by various names such as ‘lateral epicondylitis’ or ‘tennis elbow’, but the currently accepted description is ‘lateral elbow tendinopathy’ (LET) [2]. The condition has a high economic burden [3, 4] and, as no universal consensus guidelines for treatment exist, is a topic of continued interest to researchers. A wide array of interventions have been studied including surgery, numerous injectate formulations, electrotherapies and exercise. The goal of these interventions is to restore, improve or preserve function and well-being, with the effect of the intervention quantified by the patient using standardised measurement instruments. It has been recognised that there is substantial heterogeneity of instrument use in elbow pathology and specifically for LET [5]. With no clear consensus on which instruments most accurately represent a patient’s LET-related health status, comparison of effectiveness research and evidence synthesis/meta-analysis has been significantly hampered. Without clear consensus on which outcome measure best reflects the patients’ experience of LET, it is likely that agreement on treatment protocols will remain unattainable [6, 7].

In 2019, a working group of international experts in the field of tendinopathy (International Scientific Tendinopathy Symposium Consensus (ICON) Group) comprising researchers, health care professionals and patients published a consensus document defining the core domains of patient and clinician interest in tendinopathy, against which pathology-specific outcome measures should be recommended [8]. The robust methodology involved a scoping review of tendinopathy research papers, published during a 10-year period, to identify measures used and to produce a base list of 24 possible domains representing all facets of a patient’s life that may be affected by the condition. A three-stage Delphi process was then conducted involving experienced clinicians, researchers and patients from across the world to establish a consensus on which domains should be included in a core outcome set.

The nine domains that were recommended are as follows:

1. Patient rating of condition (a single assessment numerical evaluation)
2. Participation in life activities (day to day, work, sport)
3. Pain on activity/loading
4. Function (a patient-rated measure of function not relating to the intensity of pain)
5. Psychological factors
6. Physical function capacity (including strength)
7. Disability (composite scores of pain and pain-related disability)
8. Quality of life (general well-being)
9. Pain over a specified time

Numerous methods exist to measure each of these domains, so the proposed next step is to identify the suitable measures of these domains within a pathology-specific context for each of the major tendinopathies. In keeping with good practice for instrument selection, each instrument will need to be both practical to perform (based on cost, patient burden and availability) and of high quality (valid, reliable, acceptable and interpretable) [9]. The result will be a minimum set of outcome measures for each specific condition to be used in future research that allows direct comparison between different studies across the nine domains. The Core Outcome Measures in Effectiveness Trials (COMET) Initiative provides guidance on this process and a register of specific projects (http://www.comet-initiative.org). Following this guidance, the aim of this paper is to outline the protocol to be used in the development of a patient, clinician and clinician focused core outcome set (COS) for lateral elbow tendinopathy, although a similar methodology might be applied to other tendinopathies in the future.

## Scope

This COS relates to all adults diagnosed with LET and applies to interventional research (including surgical and non-surgical) and longitudinal assessment. The COS will only apply to the English language.

## Method

This study will utilise an international group of patients, researchers and clinicians to reach a consensus on the core outcome set for LET assessment. The project is registered with the COMET Initiative: http://www.comet-initiative.org/Studies/Details/1497, and this protocol is written following the Core Outcome Set Standardised Protocol (COS-STAP) checklist [10].

### Study design

The COS-LET development will progress through three distinct phases: (1) an update of the 2019 systematic review assessing patient-reported instruments in the assessment of LET will be undertaken [11], this will aim to identify additional outcomes and to gather data on non-patient reported instruments utilised in LET assessment; (2) domain matching of the individual instruments to the core domains of tendinopathy, the aim will be to ensure comprehensive domain coverage, identify gaps in coverage and to exclude instruments that do not match to the requisite domains; and (3) an international patient, researcher and clinician Delphi Study, the aim of which will be to assess the acceptability, rationale and metric properties of the instruments, thereby resulting in a selection of instruments that could be included in the COS. Following the three development stages, an expert panel of patients, researchers and clinicians will ratify the final COS.

### Steering committee

This international committee will be comprised of eight experts in the field of LET. There will be a mix of researchers and clinicians, both from surgical and physiotherapy backgrounds. There will also be a mix of nationalities, age and sex to give a broad spectrum of views. A practical challenge will be the coordination of meetings and study tasks given the spread of geographical locations of those involved. Meetings will be scheduled using an online availability tool that accounts for different time zones (www.doodle.com). Files will be stored on a secure cloud platform (www.dropbox.com) with access to view and edit given only to the Steering Committee. Meetings will be held via an online video conference platform (www.zoom.us) with minutes documented and meetings recorded in video format. This allows the Steering Committee members unable to attend meetings to accurately review the matters discussed. The steering committee will agree on the final protocol and will be in regular contact throughout the entire process up to and including the development of the final COS. The steering committee will independently conduct stages 1 and 2 of the protocol and will remain involved in stage 3 and final ratification.

### Stakeholders and recruitment

The steering committee is committed to ensuring the COS development is conducted by a broad and representative population of researchers, clinicians and, most importantly, patients. The Delphi study population will include experienced clinicians and researchers nominated by the Steering Committee and also identified by their reputation as elbow specialists or prior publications related to LET. Additionally, a search of the Expertscape and SCOPUS databases by author and filtered by the terms ‘tennis elbow’ and ‘trial’ will identify a list of other researchers to approach. Representation from a range of nationalities, with a spread of ethnicity and sex, will be ensured. Patient representation will be achieved through invitation by the clinicians on the Steering Committee, and those in the Delphi group will be asked to share the survey with their patients. A target number of 22 clinicians/researchers and 5 patients will be included.

#### Phase 1: Systematic review—instruments currently employed and their prevalence of use

In 2019, Evans et al. published a systematic review of English language outcome measures used in surgical and non-surgical trials for LET, but only instruments related to elbow pain and function were included [11]. The study reported on the psychometric assessment of 15 patient-focused instruments in LET, reported in 105 articles. The published findings and unpublished additional data, with permission of the lead author, will be used to identify all LET trials up to 2017. The searches will be repeated to include papers from 2017 onwards to subsequently provide a comprehensive list of all trials related to LET (search strategy available in Supplementary File 1 from the original publication https://tinyurl.com/y5n667be) [11]. The previously utilised search strategy constructed using MeSH and free-text terms will be run in MEDLINE and Embase, accessed through the OVID Silver Platter. The search results will be screened initially by title and abstract by two reviewers independently of each other using the online Rayyan tool to facilitate this process being completed in different geographical locations [12]. Any points of disagreement will be discussed and reconciled with the help of a third reviewer if required. All study methodologies will be included with the exception of research protocols, case studies and small case series of less than five patients as these will add little value. From the extracted lists of the 105 previously identified articles, and articles identified from 2017 onwards, a de novo data extraction will be undertaken. Extracted data will include all outcome instrument used (including patient-reported, clinician-reported, LET-specific, upper limb- or joint-specific, generic and physical examination (e.g. range of motion or strength) instruments), number of patients included in the study and full details of any novel instruments.

The output from phase 1 will be a comprehensive list of all reported outcome instruments and their prevalence of use within the entire body of literature on LET.

#### Phase 2: Domain mapping

The eight Steering Committee members will map the individual instruments to the nine core tendinopathy domains [8] using the Outcome Measures in Rheumatology (OMERACT) truth (part a) and feasibility filters [13]. The truth filter (part a) assesses the content and face validity whilst the feasibility filter assesses whether the instrument is practical to use (for example, is it too time-consuming, costly, requiring specialist equipment?). For each filter, a traffic light rating is applied. A green light means the instrument is included. An amber light means it is included with caution. A red light means it should be excluded. At this stage, if any instrumentis rated red, it will be excluded. The Steering Committee will split into four pairs to divide the workload. Each individual will assess whether their allocated instruments map to any of the nine tendinopathy domains using the instrument’s published development article or manual as a reference guide [8]. The results will then be compared with their co-reviewer and any points of disagreement discussed. In cases where a conclusion cannot be agreed, a third member will cast a deciding vote.

The outcome from phase 2 will be a list of domain-mapped candidate instruments that demonstrate adequate content and face validity and are deemed feasible for use. All instruments that do not pass these requirements will be excluded at this stage.

#### Phase 3: Delphi consensus

Following the collation of a list of candidate instruments, an international multidisciplinary Delphi study will be performed. An online Delphi questionnaire will be shared with the stakeholder group through the Qualtrics survey software (Provo, UT, USA). The Delphi questionnaire will contain a participant information sheet and consent filtering questions (i.e. ‘Yes’ is checked to consent and proceed to the questionnaire; ‘No’ is checked to not consent and be exited from the questionnaire). The Delphi questionnaire will list each instrument available for each of the nine domains with an associated reference document including the instrument’s original development article and/or manual. Respondents will rate each instrument using the OMERACT traffic light system for truth and feasibility, as for validity and feasibility. Additionally, they will be asked their opinion as to whether each instrument should be included in the final COS. Any instruments that are rated of limited importance (red) by 70% or more respondents will be excluded. This threshold is in line with the ICON Group consensus utilised for the development of the core domains [8]. There will also be the option at this stage for respondents to list any other instruments that have not previously been included if they think that inclusion is important or critical. These instruments will then be scrutinised by the Steering Committee using the OMERACT truth (part a) and feasibility filters.

Following the exclusion of instruments of limited importance and inclusion of any new instruments, the OMERACT truth (part b) and discrimination filters will be applied with a focus on the measurement properties of the instruments. Specifically, this will assess each instrument on the strength of its associated psychometric properties, as reported in development or validation articles. The metric properties that will be reviewed include the construct validity (does the instrument measure what it purports to measure, demonstrated through correlation with gold standard or associated instruments), its reliability (that the items of the instrument are coherent within its specified target domain, quantified by Cronbach’s *α*), repeatability (its stability on repeated testing with no clinical change, quantified with test-retest reliability), the instrument responsiveness (does it change with clinical change, often quantified by effect size) and interpretability (its ability to differentiate between groups, often represented by the minimal important difference). This will be done using an appropriate method, such as the EMPRO tool [14], by pairs of reviewers from the research team, and a third reviewer will cast a deciding vote in the case of disagreement. Again, these will be rated using the OMERACT traffic light system.

The Steering Committee will then compile a report summarising the findings of round 1 of the Delphi study showing the traffic light rating of each instrument within their associated matched domain and subsequent outcome of the truth (part b) and discrimination filters. The stakeholder group will then be invited to participate in the second round of the Delphi study. They will be asked to read the summary report and re-rate each instrument using the OMERACT traffic light system. The responses will be analysed, and those instruments rated of low importance (red) by 70% or more of stakeholders will be excluded unless they are the only measure of a specific domain. For each domain, instruments rated green by 70% or more of stakeholders will be included in the final COS unless there are more than one selected for that domain. In the scenario of multiple appropriate measures covering a single domain, a decision will be made following discussion in the third-round Delphi consensus meeting. Similarly, if a domain has no identified instruments, then those rated amber will be will enter the third-round Delphi consensus meeting. The online surveys are expected to take no longer than 60 min to complete and can be undertaken over several sessions.

The third-round Delphi consensus meeting will be held online via Zoom to maximise participation from clinician, researcher and patient stakeholders. Prior to the third-round Delphi consensus meeting, semi-structured qualitative interviews will be conducted with patient contributors by the research team to ensure that the patient voice is heard, due to concerns that patients may find voicing their opinions in a group environment intimidating. The anonymised findings will be used to prompt discussions in the Delphi consensus meeting. Following discussions, a final ratification process will be undertaken to develop the COS-LET. If a domain has a single instrument with green consensus agreement, this will be included in the COS-LET. For any domain that does not have a single green measurement instrument, there will be a final vote on which instrument to include. This will be done using a nominal group technique using a 70% threshold for consensus. Voting will be done anonymously using an online polling system. For domains where there is no instrument rated green with 70% consensus, we will make recommendations for research to determine a measure and recommend an instrument that may be used in the interim.

**Fig. 1:**
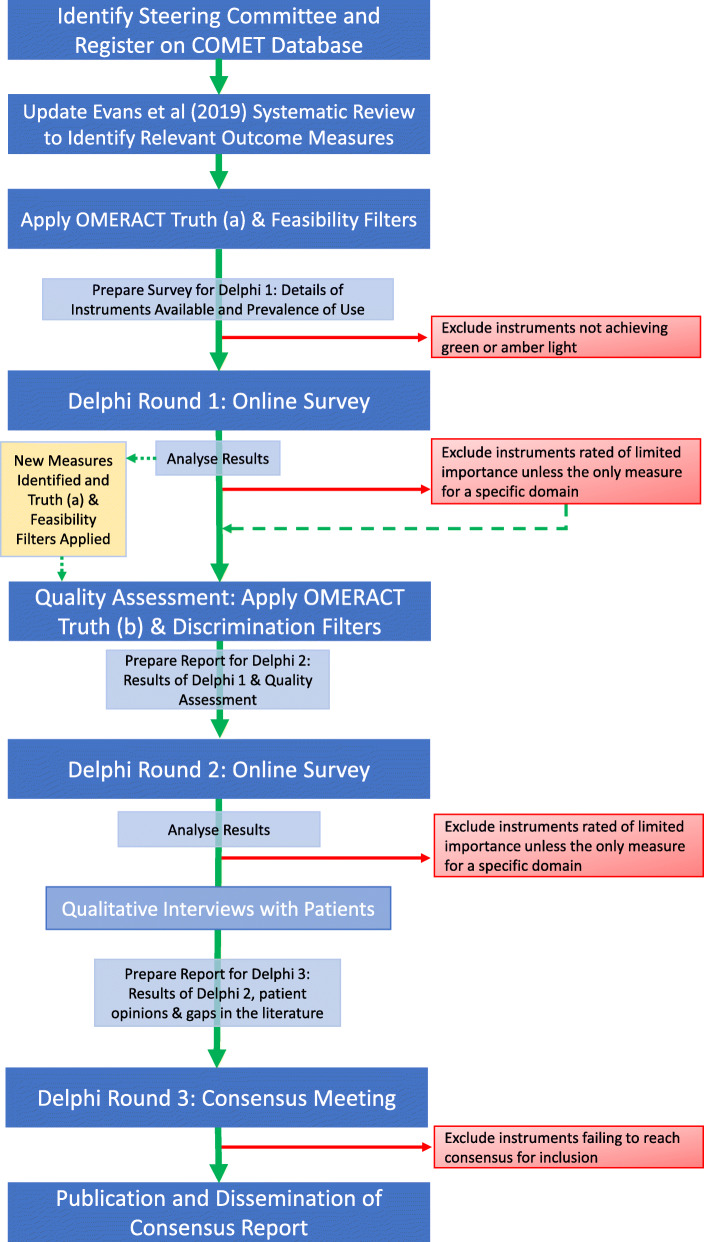
Study flowchart

A diagrammatical summary of the method is shown in Fig. 1.

### Delphi study sample size

The question of participant numbers is dependent on the minimally sufficient number to constitute a representative pooling of judgements [15]. Wide variations in expert numbers have been reported in Delphi studies [16], though nominally they tend to be within 20–60 [17].

This study will aim to recruit a minimum of 30 participants. The primary steering group from phases 1 and 2 will comprise eight members. Therefore, an additional 22 clinician/researcher participants will be required during the phase 3 Delphi study; however, there will be no maximum. At least 5 patient participants will also be included.  Participant retention is an important component of Delphi studies; therefore, the iterations of the Delphi will require retention of >60% of respondents. Though it has been reported that the reliability of composite judgements increases with respondent numbers, there is little empirical evidence on the effect of participant numbers on the reliability or validity of the consensus process if the panel composition is appropriate [18].

### Final development

The goal of this body of work is to achieve a pragmatic COS that will be readily employable. Our attention will be set to the production of a COS that achieves a clear patient focus, representing the outcomes important to them. It should be comprehensive but also user friendly, wherein there is minimised burden both to the participant completing the outcome scores and the researcher in its delivery and interpretation.

### Ethical considerations and data collection

In concordance with the principles of Good Clinical Practice (GCP), phases 1 and 2, whereby the committee membership undertake non-patient-facing activity utilising previously published literature, that formal ethical approval will not be required. For the Delphi study, ethical approval has been granted by the University of Queensland research ethics committee (reference number 2020001340).

All data will be handled confidentially and in accordance with the General Data Protection Regulation (GDPR). Access to any personally identifiable data will be strictly limited.

### Dissemination

The final report detailing the instruments selected in the COS will be submitted for publication in a high-quality peer-reviewed international journal and will be presented at relevant international scientific meetings such as the International Scientific Tendinopathy Symposium and International Congress of Shoulder and Elbow Surgery. The key points, infographic and links to the final report will also be disseminated via social media platforms.

## Results

Not applicable for a protocol article.

## Discussion

This protocol has been designed to comply with contemporary standards and expectations of a core outcome set development; however, the authors recognise that certain limitations are inherent in an outcome study design of this formulation.

### Outcome selection

Within the systematic search component of phase 1, the search strategy development is guided by previously published search strategies for systematic reviews of interventions in elbow pathology [19] and for the identification of outcome measures [20], along with terms specifically selected in order to capture names of relevant instruments published in previous systematic reviews of elbow-specific rating scales [21–24]. Although the intention is that this is as sensitive as possible, it remains a possibility that instruments will not be identified through this process. All stakeholders will be consulted on whether they are aware of further measures that should be included.

To assist in the running of the Delphi study, the list of included instruments will be honed as part of the domain matching process, wherein all unmatched instruments will be removed. This process will rely on the face validity assessment of the instrument by two reviewers and risks unwarranted exclusion of instruments. Again, to ameliorate this risk, the steering committee will have oversight on the included and excluded measures.

### Stakeholder selection

Careful consideration has been given to the composition of both the steering group and larger stakeholder group selection. Efforts will be made to invite a broad and representative selection of contributors from an international multidisciplinary pool including a patient sample. However, the authors recognise that certain groups may be unrepresented or omitted. The anonymity of the Delphi study process will assist in the protection of bias from the influence of certain factions of a stakeholder group, but it remains a possibility that certain sub-groups may hold more influence. To monitor this risk, the steering group will maintain oversight, and a stratified analysis of the Delphi results will be undertaken to assess for skewing.

### Consensus population

The decision to restrict the COS-LET to English language use is an inherent limitation in its utility; however, this decision has been made on a pragmatic basis. Restriction of language eligibility will allow a clearer communication between the steering and stakeholder groups and will mean that the Delphi study can be delivered in a single language. We also recognise that outcome instrument cross-cultural validation necessitates a meticulous methodological undertaking in itself [9], therefore to recommend instruments applicable across language becomes very challenging and beyond the scope of this protocol.

### Outcome measures

The output from this study will be a recommended COS of instruments. However, if particular domains are covered equally well by multiple instruments, a decision may be made to allow the recommendation of multiple instruments for use at the discretion of the future user. It is also beyond the scope of this work to designate which of the COS-LET instruments should be primary outcomes or at which time points they are to be collected. These aspects will be discussed in depth within the final article.

## Conclusion

This protocol describes the methods that will be applied in the development of the core outcome set for lateral elbow tendinopathy (COS-LET). A rigorous approach that complies to the standardised expectations of the core outcome set development has been proposed. The aim will be the production of patient-focused, user-friendly and comprehensive guidance on outcome set selection with the goal of broad implementation into future LET research.

## Data Availability

This protocol has been registered with the COMET initiative. Full data transparency will be provided with the publication of the final study findings.

## Abbreviations

COMET: Core Outcome Measures in Effectiveness Trials
COS: Core outcome set
COS-STAP: Core Outcome Set Standardised Protocol
GCP: Good Clinical Practice
GDPR: General Data Protection Regulation
ICON: International Scientific Tendinopathy Symposium Consensus
LET: Lateral elbow tendinopathy
OMERACT: Outcome Measures in Rheumatology
